# Local constraints to access appropriate malaria treatment in the context of parasite resistance in Cambodia: a qualitative study

**DOI:** 10.1186/s12936-017-1732-0

**Published:** 2017-02-17

**Authors:** Jesse Verschuere, Tom Decroo, Dara Lim, Jean-Marie Kindermans, Chea Nguon, Rekol Huy, Yasmine Alkourdi, Koen Peeters Grietens, Charlotte Gryseels

**Affiliations:** 1 Médecins Sans Frontières, Operational Centre Brussels, Phnom Penh, Cambodia; 2 Médecins Sans Frontières, Operational Centre Brussels, Medical Department, Brussels, Belgium; 3 Center for Parasitology, Entomology and Malaria Control, Phnom Penh, Cambodia; 4 Medical Anthropology Unit, Department of Public Health, Institute of Tropical Medicine, Antwerp, Belgium

## Abstract

**Background:**

Despite emerging drug resistance in Cambodia, artemisinin-based combination therapy (ACT) is still the most efficacious therapy. ACT is available free of charge in the Cambodian public sector and at a subsidized rate in the private sector. However, un- and mistreated cases in combination with population movements may lead to the further spread of resistant parasites, stressing the importance of understanding how the perceived aetiology of malaria and associated health-seeking behaviour may delay access to appropriate treatment. A qualitative study explored these factors after an epidemiological survey confirmed parasite resistance in Preah Vihear province.

**Results:**

In Cambodian cosmology, illnesses can be inflicted by supernatural beings or originate from ‘natural’ causes because of disorder in the social, domestic or outdoor environment. Initial treatment options consist of cheap and accessible home-based care (manual therapy, herbs and biomedical medication) targeting single symptoms. If there is no steady recovery or if the condition quickly aggravates, care will be sought from ‘village doctors’, public health facilities, private pharmacies or, in case of suspicion of a supernatural cause, from a specialized indigenous healer. The choice of provider is mostly based on the family’s financial situation, access to and trust in the provider, and the congruence between the suspected aetiology of the illness and the treatment offered by the provider. Different treatment options are often combined during the same illness episode through a serial process of trial and error guided by the observable improvements in the patient’s condition.

**Conclusions:**

Cambodian perceptions of illness that focus on single symptoms and their perceived severity may lead to the identification of one or multiple illnesses at the same time, rarely suspecting malaria from the start and implying different patterns of health seeking behaviour and treatment choice. However, decisions to self-diagnose and treat at home are also pragmatic and must be understood in the context of poverty, a major barrier to seeking prompt and appropriate care for malaria in an area characterized by parasite resistance.

## Background

Although there has been a steady decrease of malaria cases in Cambodia during the last years, the malaria parasite’s resistance to artemisinin remains an important factor of concern [1, 2]. In biomedical discourses, drug resistance is explained by genetic mutations in malaria parasite populations that are favoured due to repeated exposure to subcurative or substandard doses of anti-malarials [3–5]. Scientists agree that malaria parasite populations under drug pressure can evolve through the *use* of those drugs by people, exposing the social dimension of this biological event. Along the Thai-Cambodian border, resistance of the *Plasmodium falciparum* parasite to chloroquine emerged about half a century ago, followed by resistance to the drugs sulfadoxine-pyrimethamine and mefloquine [1, 6–9]. More recently, reduced sensitivity to artemisinin and artemisinin-based combination therapy (ACT) have been discovered in the same area [9–11]. Converging socio-economic forces may in part explain the first emergence of chloroquine resistance in the 1950s: at that time, a high influx of migrant mine workers were being exposed to sub-therapeutic dosages of prophylactics, and in combination with the mosquito’s ability to thrive in the mine shafts, this might have contributed to the survival of resistant parasite strains [4, 12, 13].

Nowadays, high human mobility [14, 15], low access to and quality of public health services [16–19], and the popularity of a mostly unregulated informal private health sector [17, 19–22] still characterize the social landscape in Cambodia, potentially contributing to the ever growing and spreading drug resistance of the parasite. Despite reduced sensitivity to ACT, this therapy is still the most efficacious treatment available, and is provided to the Cambodian population free of charge or at subsidized prices through Village Malaria Workers [16], public health centres and hospitals, and private pharmacies. However, although they are now officially banned in the private sector, evidence suggests mono-therapeutic anti-malarials of which the quality is uncertain are likely still available, potentially contributing to the mechanisms underlying parasite resistance [19, 21]. Moreover, mistreated cases in combination with a highly mobile population—forest goers, plantation workers, military personnel, etc.—may lead to the further spread of resistant parasites [14, 15]. In this context of growing parasite resistance, it is important to understand the socio-cultural dynamics underlying the use of medicines, as the perceived aetiology of malaria and the associated health-seeking behaviour may delay access to appropriate treatment, resulting in un- or mistreated malaria cases.

Following the commitment of Médecins Sans Frontières (MSF) to support the Ministry of Health in the elimination of *P. falciparum* in Preah Vihear province, a cross-sectional population-based malaria survey was organized in 2013 in Chhaeb and Chey Saen districts in the north-east of the province, which confirmed the presence of the *k13*-propeller gene (*k13*) mutation in the *P. falciparum* parasite associated with artemisinin resistance [23]. Because of varying levels of artemisinin resistance and in order to stratify containment activities, the WHO recommended to classify areas in endemic countries according to three tier-levels. In areas labelled as Tier 1, such as Preah Vihear province where this study was conducted, there is credible evidence of artemisinin resistance for which “immediate, multifaced response” is recommended [24]. It was considered important to understand the social dimension of artemisinin resistance, which includes understanding the perceived aetiology of malaria and the associated health-seeking behaviour in order for any subsequent measures to be effective. To this end, a qualitative study was conducted ancillary to the cross-sectional malaria survey, of which the results are presented here.

## Methods

### Study site and population

**Fig. 1:**
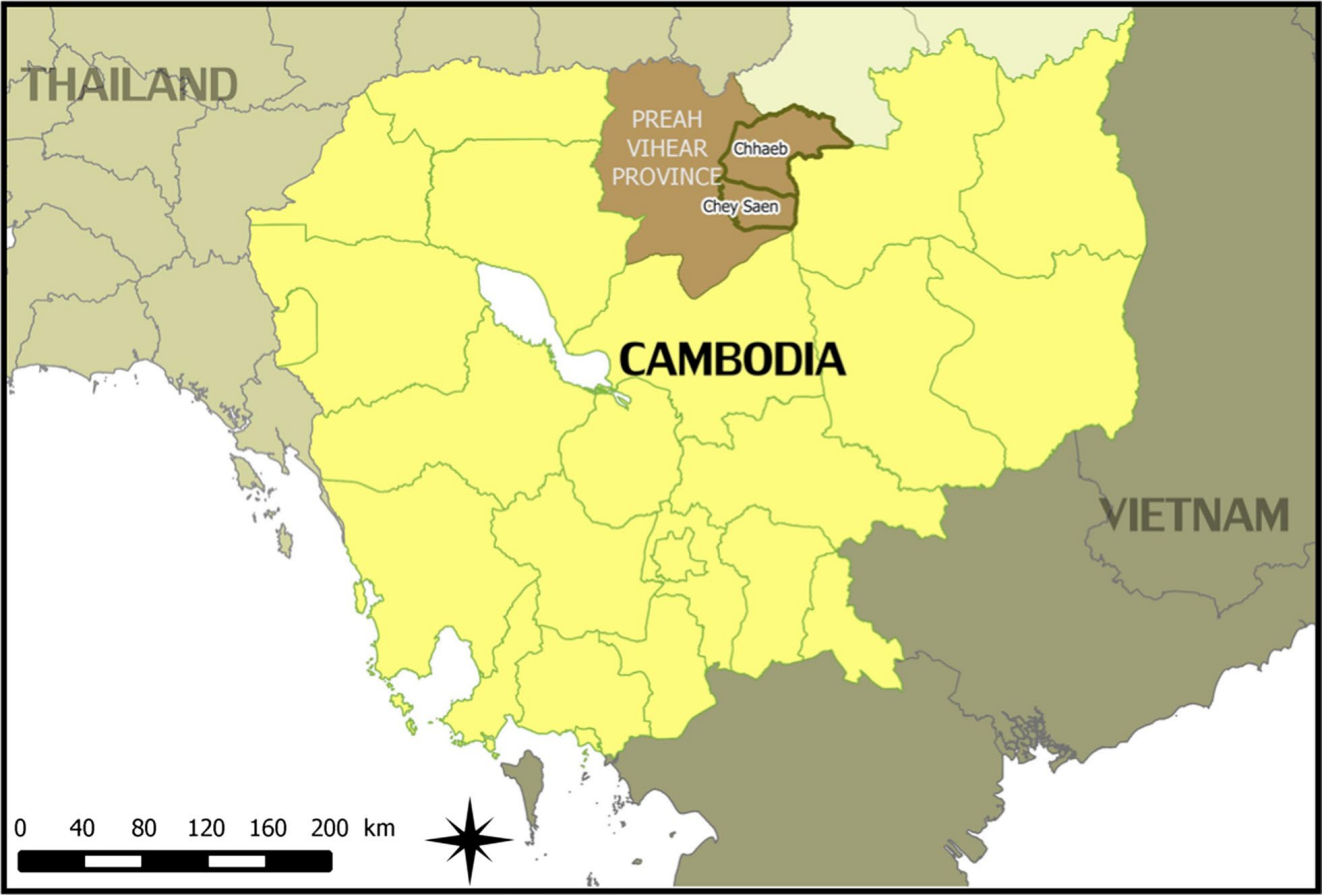
Map of Cambodia locating study area

The study took place in Chhaeb and Chey Saen districts in Preah Vihear Province, bordering Thailand and Lao PDR (see Fig. 1). Large parts of the province are remote and heavily forested despite the ongoing deforestation. The province has poor infrastructure and in the rainy season access to villages is difficult. The area is mainly inhabited by Khmer (dominant population in Cambodia), Kuy (Cambodian ethnic minority) and Lao people. International migrants, i.e. Chinese workers active in construction, mining or plantation, as well as domestic migrants, i.e. Khmer from other provinces in search of work and better living conditions, have also moved to the province.

### Data collection and sampling

The study took place from August to November 2013 and used research techniques associated with focused ethnography, including participant observation, in-depth interviewing and focus group discussions. Participant observation consisted of daily life observations and reiterated informal conversations during field stays in the study villages. This technique was used to detect unforeseen variables and to contrast stated opinions with actual behaviour, constituting a respondent independent data collection tool. In total, 42 in-depth interviews have been conducted in Khmer, using a topic guide, a trained note taker and a translator. In addition, 7 focus group discussions were done with male and female villagers separately. Each interview and focus group discussion was audio-recorded, transcribed in Khmer and translated into English. For in-depth interviews and focus group discussions, participants were purposively selected, aiming for maximum variation based on four selected key demographic variables: gender, age, ethnicity, provenance and literacy. Key-informants were theoretically selected in accordance with preliminary theories that were developing based on intermittent analysis of the data. When no new useful information emerged from the data and saturation was reached, data collection stopped.

### Data analysis

Following the principles of grounded theory, analysis of the qualitative data was an inductive iterative process, where data was repeatedly explored, interpreted, coded and categorized. Data from different sources was triangulated to test the consistency of the findings. Nvivo 10 Software (QSR International Pty) was used to analyse the data.

## Results

### Study participants

19 key informants, theoretically selected among medical staff, village chiefs, village malaria workers, traditional healers, private health providers, and seasonal workers, were interviewed (Table 1). Another 23 in-depth interviews were conducted with 26 community members (in 3 households, 2 household members were present during the interview). The 7 focus group discussions included 49 participants in total (Table 2).

**Table 1:** Profile of key informants

Occupational profile	N
Private health providers	4
VMWs	2
Traditional healers	4
Community leaders	4
Public health staff	3
Seasonal workers	2
Total	19

**Table 2:** Participant characteristics

Participant characteristics		IDIs	FGDs	Total
Gender	Male	10	26	36
	Female	16	23	39
Age	15–30	9	12	21
	30–45	6	20	26
	45–60	11	17	28
Literacy	Literate	18	16	34
	Illiterate	8	33	41
Ethnicity	Khmer	39	26	65
	Kuy	6	23	29
Provenance	Chhaeb	15	31	46
	Chey Saen	11	18	29

### Illness aetiologies

In Cambodia, illness is perceived to originate either from the supernatural realm, inhabited by deities and spirits, or from ‘natural’ causes—and sometimes by a combination of both. In all cases, the manifestation of illness is perceived to be inseparably linked to the presence of ‘disorder’. Order is connected to cleanliness and appropriate moral behaviour, and disorder—often produced by a lack of hygiene or by social transgressions—is perceived to lead to disease.

### Supernatural realm

Demons, spirits or deities can be provoked to inflict illness by “bad” behaviour, which means a person or family causes social disorder by fighting, disrespecting elders, intra-familial arguments, etc.

*R: Sometimes the [malaria] patient says it is maybe a ghost that attacked [him], such as the deity of the forest or mountain!*

*I: And for the case of your grandchild, she just got sick for 2 or 3* *days and then she died?*

*R: Yes, after two days she died. […] Some people said she was defeated by the ancestral spirit, she had convulsions and said something without thinking.* (Interview with a middle-aged male farmer)

Through sorcery (*ampeu)*, human individuals can also access supernatural powers that can cause illness. When someone does something wrong or misbehaves towards another person, that person may request a sorcerer (*thmorb*) to inflict disease on the social transgressor. The sorcerer may use magical spells and curses, or magically insert objects such as nails, needles, little bones or bits of buffalo skin into the body of his victim. Another way to be attacked by sorcery is to accidently step on or walk over a *sey*-*mar*, a place or a thing that a sorcerer can invest with magical power.

*People say: ‘a person who is useless and [has] fights with others, risks to [be attacked by] ‘ampeu’ (i.e. sorcery)’. But as I’ve never started problems with anyone […] no one has ever done [sorcery] against me. […] If we do something wrong to them, they will do [it] back to [us].* (Interview with a young male farmer)

### Natural causes of illness

Illnesses can also have ‘natural’ causes, which do not only refer to microorganisms in the body. First, excessive work, sudden changes in the weather or eating the “wrong” types of food may cause disorder in the body.

*Sometimes, [when] we get better, we [have to] follow a diet. If we don’t follow the diet [we] will be sick again. For ‘krun chan’ (malaria) people cannot eat tamarind and papayas; both mixed papayas and sour papayas. [We] cannot eat noodles either. If we don’t follow this diet and still eat everything, we will get sick again, [but then it will be] more serious*. (Interview with a middle-aged female farmer)

Second, disorder in the domestic environment is also reported to be an important risk factor for contracting illness: properly arranging the things inside the house and keeping surroundings tidy are just as important as using a mosquito net or drinking boiled water on a daily basis.

*[…] if the ‘dey low’ (i.e. the land on which the house is built) of each of us [has] [puddles of] water, like these ones there, [as we] don’t take time to clear it, [they will become] shelters of mosquitoes, isn’t that right? […] As mosquitoes have a lot of places to stay, the ‘krun’ (fever) [can happen] anywhere. [We have to] clean the whole house and its surroundings*. (Interview with a middle-aged male farmer)

Third, external disorder, which is inherent to places such as the forest, is also perceived to be a risk factor for illness, as multiple respondents reported malaria to be caused by spending too much time in the forest.

*During the first years, when I just arrived here, I never went into the forest, [so] I never had ‘krun chan’ (malaria). [But since] the last three, four years, when I went to work on the farm, [I] began to [have] ‘krun’ [chan] (malaria).* (Interview with a middle-aged female farmer)

### Double causality

Informants report that in the past, people in Chhaeb and Chey Saen believed that someone had *krun chan* (Khmer for ‘malaria’) because they had offended the deity of the forest. Nowadays, almost everyone considers the mosquito bite to be the primary and natural cause of the *krun chan* illness. However, informants explained *krun chan* may also have an underlying supernatural cause, such as an angry ancestor spirit or deity that influences the mosquito or the parasite causing the natural disease. When treatment does not result in recovery or when the disease aggravates substantially, people may experience this as proof that the illness is actually caused by something ‘invisible’ (*vea mean ey na*).

*It is like my younger sister, last month. […] As [she] had convulsions*—*[her] eyes were rolling upwards, [her] mouth was twisting*—*my parents became very worried about [their] child, so [they] asked [the medical staff] to take [her] out of the hospital. […] [They] went straight to the […] indigenous healer in the provincial capital. […] He said she was possessed by 3 or 4 ancestor spirits. We did the ‘saen’ (the offering for the spirits) and they left her body. She recovered and survives until this day.* (Respondent in a focus group discussion with female farmers)

### Illness perceptions

The descriptive language Khmer people use to explain their health condition is based on a construct of illness that focuses on symptoms or body parts. When referring to a specific illness, the combination of one of the Khmer words for ‘illness’ (*krun, cheu, jum*-*gneu)* and the Khmer word for the symptom or the body part causing the discomfort signifies the situation at hand. For example, *krun pos vean* means illness (*krun*) related to the intestines (*pos vean*), *cheu kraw peah* illness (*cheu*) related to the stomache (*kraw peah*) and *cheu kbaal* illness (*cheu*) of the head (*kbaal*). In practice this implies that when a person is showing several symptoms of malaria, such as fever, headache and muscle pain, this condition will first be referred to and be interpreted as a combination of different illnesses occurring at the same time. Fever can even be further subdivided into different illness descriptions: having *krun k’daw* is related to being hot (*kdaw*), while someone who has *krun njeak* will be shivering (*njeak*) and feeling very cold.

Alternatively, an illness could transform or progress into another type of illness, as explained by a woman in Chey Saen district: “First my feet felt cold and then it became an intestinal illness (*krun pos vean)*. Later on, it became malaria (*krun chan).*” During the initial phase of malaria, symptoms are rarely diagnosed as *krun chan*—the official translation of malaria—but rather as *krun kdaw, krun njeak* or *cheu kbaal* and will usually be treated at home based on discussions with family members.

Even when the severity of the single symptoms finally results in the suspicion of malaria, people may diagnose either *krun chan* (meaning ‘the disease that defeats you’, referring to malaria), *krun vivak* (a Khmer variation on vivax malaria), *krun che* (closely linked to *krun cheam,* referring to dengue), and sometimes even *krun andeuk* (the ‘turtle disease’ which in the past was sometimes used to refer to malaria). Different health-seeking behaviours are implied in the diagnosis of these different malaria’s, although informants did not report on any uniform pattern.

*R1: Now, [after] my nephew had a blood test, [he] didn’t have ‘krun chan’, but ‘krun vivak’. […] Then [the village malaria worker] asked [us] to go to the referral hospital in the provincial capital. If it is only ‘krun chan’ or no serious ‘krun chan’, [we can] recover by the treatment provided in the district, but when it is ‘krun vivak’, they won’t allow [us] to keep [the patient here]. They send us to the referral hospital.*

*R2: If it is ‘krun chan’, [they will] call the ambulance to take [the patient]. If [it’s] ‘vivak’, [we will] buy medicines here.*

*R3: If [it’s] ‘chan’ and ‘vivak’ together, they cannot give treatment here, [so we have to] take [the sick person] to the referral hospital.*

*R4: If [someone has] ‘krun chan’ from the start, [he can] get better [easily], but now [people] mostly have ‘krun vivak’ or ‘krun che’, [so] it is not easy to get better.* (Four respondents in focus group discussion with female farmers)

### Treatment options for malaria

#### Home-based self-treatment

Home-based care methods, such as manual therapy, herbs and biomedical medication are perceived to be cheap and directly accessible and give the patient the opportunity to remain in familiar and comfortable surroundings. These methods are first choice for most respondents when confronted with initial and/or mild illness symptoms (see Table 3). Manual therapies for fever include showering, fanning (*bork*), wiping or covering the person with a *krama* (traditional scarf) soaked in water or with slices of winter melon (*traw*-*larch)*.

**Table 3:** Malaria symptoms and related local treatment options

Condition	Treatment	Examples of local treatment options
*Krun chan* (malaria)	*Tnam Khmer* (herbal medication)	Take twigs of a *sdaw* tree, peel them, mix with three cups water and boil until one cup remains, resulting in a very bitter mixture. A variant is to soak the twigs in water instead of boiling, although the mixture is then believed to be less effective Eat 10–20 chillies per day as prevention to *krun chan* Take *bon*-*dole*-*pek* (a kind of winder), the bark of the *sdaw* tree and the bark of the *kdol* tree. Cut it into pieces and clean it with water. Then you can either boil it or soak it in water, before drinking. If you boil it, you take tree cups of water and boil it until one cup is left. Repeat this procedure for another two times, adding water each time. Then add rice flour (*msaw*) to the mixture, stir it, and when it is well mixed, make round pills of it to swallow. To make these pills, often a bamboo layer is used
	*Tnam Pet* (biomedical medication)	Paracetamol (*para*) Antibiotics such as amoxicillin and ampicillin Antimalarials such as artesunate-mefloquine (*malarine*), quinine, chloroquine, tetracycline Often sold in a ready-made mix (*psom tnam*)
*Kjol* (discomfort)	Manual therapy	Coin-rubbing (*kos kjol*): a very popular self-care technique among the Khmer, during which neck, chest, back shoulders and upper arms are vigorously rubbed with the edge of a coin, spoon or cover of a small tin box, lubricated with Tiger Balm or other types of heat rub, until the scraped areas turn red. The darker the red, the more effective the procedure is perceived to be Cupping (*chup kjol*): another practice to relief internal pressure linked to *kjol*, which refers to placing heated small cups on the part of the body that is troubling the person. This can be the chest, the back, the upper arms or the forehead. Bad air (*kjol*) is perceived to be sucked out of the skin when the air inside the cup cools down and a vacuum is built. Red circular marks are left on the skin, which will remain for several days (see Fig. 2) Pulling hair strand (*dork sork*), all around the head, by short twitches, causing a feeling of svaang (relief) *Chaab kjol*, a specific type of massage which is considered as the most painful of all the techniques to relieve the bad air, as it produces bruising, by pinching and releasing skin folds repeatedly over the same site while applying Tiger Balm, thereby increasing the circulation of blood to the area
*Kob sorsaai* (blockage of meridian)	*Tnam sorsaai* (herbal medication)	Take the bark of the *bom*-*pong chong*-*krom* tree and boil it Take the roots of the *sdaw* tree and *ang kroang* plant and boil these together with leaves of the *sdoal* tree in water
	Manual therapy	Massage of the *sorsaai* (*chaab sorsaai*, *kes kaay* (i.e. “dig up”) or *tver sorsaai*)
*Krun kdaw* (fever that feels hot)	Manual therapy	Showering Fanning (*bork*) Wiping or covering the person with a krama soaked in water or with slices of *traw*-*larch* (winter melon) Coin-rubbing (*kos kjol*) Cupping (*chup kjol*)
	*Tnam trawjak* (herbal medication reducing high body temperature)	Pile bitter melon leaves and *sav mov* leaves together, add sugar, and mix it with water, which results in a very better mixture Mix the roots of three kinds of plants, *sbaw plaeng, pkaah*-*yok* and *chkaeh sreng,* together and boil it with water Take the barks of the trees *kagn*-*jes* and *roh*-*lork*, and mix it with 3 cups of water. Boil it until only one cup is left. Add water and repeat this procedure for another two times
	*Tnam pet (*biomedical medication)	Mostly paracetamol *(para)*
*Krun njeak* (fever with shivering)	*Tnam Khmer* (herbal mediciation)	Take the roots or bark of the *trom kmoach* and *ampov kmao dai darch* trees and boil them in water together with the skin of a ripe coconut for around half an hour. It is also a remedy against digestion related problems.
*Cheu kbaal* (headache)	*Tnam Khmer* (herbal mediciation)	Take the leaf *traw*-*jeak chruk*, grind it and put it in water.
	Manual Therapy	Coin-rubbing (*kos kjol*)
Fatigue	Manual Therapy	Coin-rubbing (*kos kjol*)
Malaise	Manual Therapy	Coin-rubbing (*kos kjol*)

*[We] boil water [and when] it’s hot, we take it, [soak the ‘krama’ in it] and we wipe it all over the body. After, we cover [the body] for a while. [Then] we turn [the sick person on its back], we soak [the krama] again and wipe it all over the body again. […] Then the ‘kdaw’ (hotness) will leave [the body] and the body becomes ‘traw*-*jak’ (normal temperature).* (Interview with a young female farmer)

**Fig. 2:**
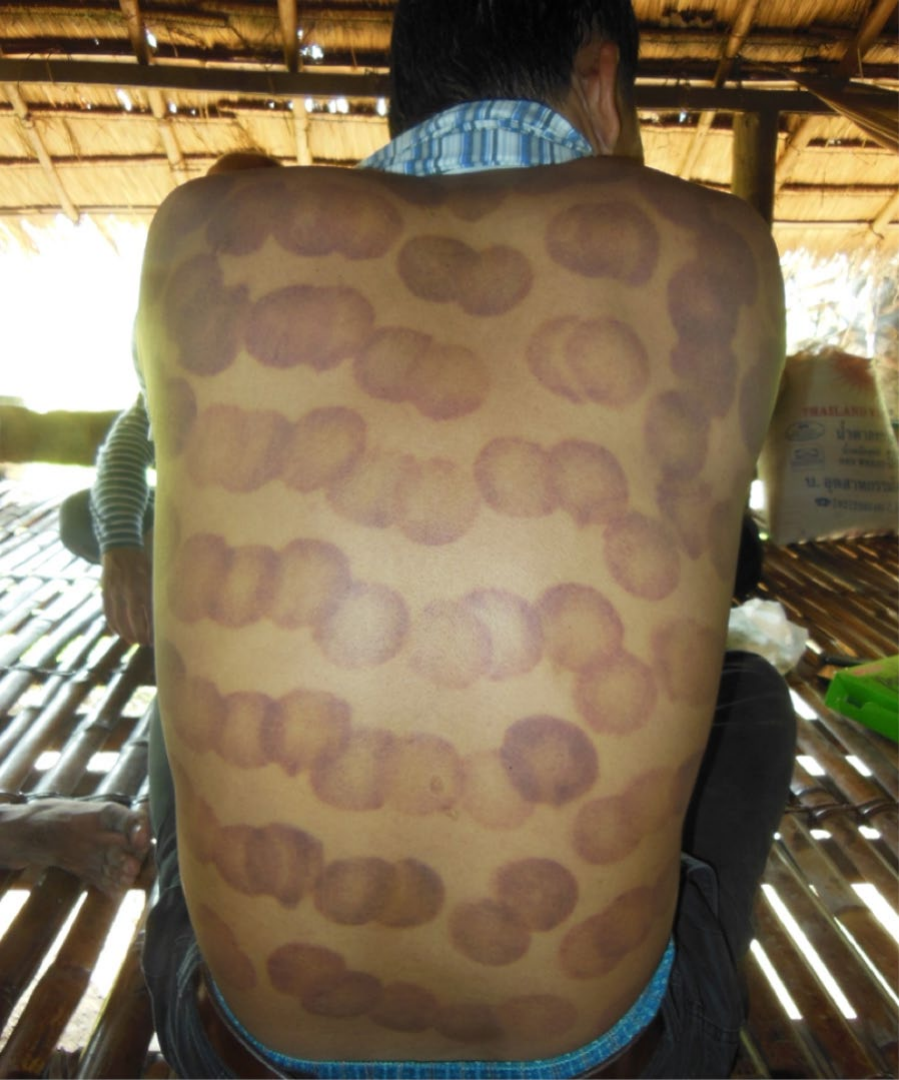
*Red circular* marks after a session of cupping

Other methods to reduce fever and to deal with symptoms such as headache, general malaise, fatigue are associated with the condition of *kjol* (general feeling of discomfort), which is perceived to be an internal pressure caused by “bad air” (Table 3). The patient has to be relieved from this pressure by coining (*kos kjol*), cupping (*chup kjol*) (Fig. 2), hair pulling and massage, thus improving the blood circulation and restoring humoral balance.

When a patient is experiencing pain, this may lead family members to suspect a blockage of the *sorsaai,* which refers to interconnected meridian, bodily conduits which transport different substances, fluids and air throughout an organism and which connect the vital organs (Table 3). A blockage of the *sorsaai (i.e. kob sorsaai or “buried” sorsaai)* may cause pain in the muscles and joints. To reduce the pain caused by such a blockage one can recur to specific herbal medicines (*tnam sorsaai*, see below), or undergo a massage of the *sorsaai* (*chaab sorsaai, kes kaay* (i.e. “dig up”) or *tver sorsaai*).

Herbal treatments (*tnam khmer*) were another important way of self-treatment for most respondents, although they were often used in combination with biomedical treatment. For almost every illness or symptom, including malaria-related symptoms, specific herbal concoctions exist based on specific plants, roots, vines or leaves. Ingredients for these concoctions are collected in the neighbouring environment (rice field, forest, gardens, etc.) by family members of the patient or provided by an indigenous healer called the *kru tnam khmer* (Table 1). Knowledge about such concoctions is widely spread and is passed down from generation to generation, although the elderly are perceived “to know best”.

Biomedical medication (*tnam pet*) is often bought for complementarity during the self-treatment phase at local village shops, drugstores or at the nearby private ‘village doctor’ (*pet phum*), where prior consultation or prescriptions are not required and payments do not always have to be made upfront and can be made in instalments, giving families time to find financial support.

*My child has had ‘krun chan’ recently. His grandmother took care of him, as he got severe ‘njeak’ (shivering). [He] was given a few ‘para’ (i.e. tablets of paracetamol) and one tablet of this [because he was] seriously ‘kdaw’ (i.e. feeling hot) and ‘njeak’ (shivering).* (Respondent in a focus group discussion with female farmers)

Most drug sellers have no formal training or pharmaceutical knowledge; they sell and combine drugs based solely on experiential knowledge and former patient reports on the perceived effectiveness of the medicines. To keep business coming their way, private drug sellers usually give clients what they ask for, which is “medicines with immediate effect”, usually referring to intravenous fluids or injections. However, pills are cheaper and are often sold in a ready-made mix (*psom tnam*), containing a couple of antibiotics, painkillers and vitamins, preferably in different colours. The extra medicines included in the bag are not perceived to be effective against *krun chan* (malaria), but help in reducing the singular symptoms the illness starts with.

The price of medication bought at the private sector, although variable, is considered high. However, as the cost of travelling is usually lower than travelling to a distant public health centre; considering there are no waiting times during which the illness may aggravate, becoming more expensive; and medication is perceived to be of better quality; visiting private providers is still often thought to be more cost-effective.

#### Biomedical treatment outside of the home

If there is no steady recovery or if the condition quickly aggravates, the illness is no longer perceived to be a regular disease that can be treated at home. The subsequent choice of treatment will first be negotiated among family members, relatives and even neighbours.

*[We] make decisions together… [It] is decided by husband [and] wife, yes. […] Sometimes, there are relatives [or] neighbours coming over to listen, to offer ideas. […] Yes, [it’s] like exchanging ideas. […] Sometimes, the person who is taking care of the illness is so worried about the child that, [he/she] doesn’t think clearly anymore.* (Respondent in a focus group discussion with male farmers)

The sick person and his or her family will search for help in the nearest public health facility, the private clinic, the local ‘village doctor’ (*pet phum*), the local village malaria worker (VMW) or the local healer. If *krun chan* (malaria) is suspected by the patient’s social network, this needs to be confirmed by a blood test (RDT) available in the public health sector or from the VMW, or in absence of a RDT, by the (private or public) medical practitioner, relying on the observation of the clinical symptoms, saying the sick person has *krun chan*.

*I don’t do the blood test, you know. I only know [someone has ‘krun chan’], when they come here to see me. This morning, [someone] came here and described the symptoms as shivering, then fever, and afterward [he] started to sweat.* (Interview with a male ‘village doctor’)

Multiple informants told us that private ‘village doctors’, who do not have RDTs available, will refer the patient to a VMW to do a blood test before providing any other treatment. Respondents stressed the distinction between pills (*tnam krop*) and the injectable medicines (ampoules for injections diluted in IV fluids) that are available in health facilities and clinics. Most informants believe that an injection (*chak*) or intravenous fluid therapy (*serum*) is more effective than taking “medicines to swallow” (*tnam leb*).

*If the pills are too slow to kill the illness, we take the child for an injection)!* (Interview with a middle aged male forest worker)

Private practitioners are more inclined to give the patient injectable therapies because they believe this is what the patient wants, therefore assuring the return of clients while at the same time making more profit than what is to be made from selling “medicines to swallow”.

*There is only one private practitioner here, that sells ‘tnam pet’ (‘biomedical drugs’). Anybody who is staying at his house, they give injections. […] An injection is more effective than pills.* (Interview with a middle-aged male farmer)

Informants made a distinction between the kind of biomedical treatment that is required for *krun vivak* (vivax malaria) and *krun chan* (malaria). However, these perceptions were sometimes contradictory. Multiple informants believed *krun vivak* to be more serious than *krun chan*: “when it is *krun vivak*, they won’t allow us to keep [the patient in the district health centre]. They send us to the referral hospital.” Other informants referred to *krun chan* as being more severe: “if it is *krun chan*, [they will] call the ambulance to take [the patient]. In case of *vivak*, [we will] buy medicines here.” (quotes from focus group discussion with male farmers)

### Counter-sorcery

If the condition of the sick person does not improve after having tried different types of treatment, informants often suspected a spiritual or sorcery-related cause, even in the case of diagnosed malaria. To cure illnesses inflicted by magical means, affected people seek the intervention of a specialized indigenous healer (*kru khmer*), a spirit medium (*kru chol ruub*) and sometimes even a Buddhist monk, as they can communicate with the spirit world and neutralize the affliction. In case an angry ancestor spirit or an offended deity causes the illness, the *kru khmer* will often advice the patient and the family to make an offering (*saen)* at the shrine in their house or at the rice field, to appease the offended ancestor spirit (*yeay taar)* or deity (*neak taar)*. Sometimes the offering to the spirits is done at the house of the *kru khmer*, by making a ceremonial tray of banana stems and leaves (*bae).* This can be followed by *sdos* (a treatment consisting of spitting), which often happens in combination with providing herbal medicines based on the symptoms of the illness.

*When I was not well, I went first to private practitioner to check my ‘kraw peah pos vean’ (illness related to the digestive system). […] I came back [home] and I took medicines, but I still couldn’t recover. I went to buy ‘serum’ (IV therapy), which was not effective at all. [I thought I] was going to die. […] [Finally, I] decided to try [with the indigenous healer]. [When I] arrived, he asked [me] to prepare a ‘bae sey’ of two or three layers (i.e. a small square tray of banana stems and leaves, often decorated with flowers and)[…], ‘sdos’ (treatment consistent of spitting) and ‘chak teuk’ (treatment consisting of pouring of blessed water) three times a day. […] Because the indigenous healer did the ‘chak teuk’ I could recover and stay alive until today. (*Respondent in a focus group discussion with female farmers)

When someone is attacked by the *roh*-*bos som*-*ngut* (‘hidden thing’, i.e. sorcery), one of the treatment methods is *leak kaab*, which means ‘to suck out the sorcery’ by using a chicken egg or a betel nut (i.e. areca nut) wrapped in betel leaf, sometimes along with limestone, and withdraw the foreign objects (cf. supra) the sorcerer has inserted in the patient’s body. Based on mutual trust, the patient actively cooperates in the therapeutic process, during which he enters in an intimate student–teacher relationship with the *kru khmer* and other family members are usually present.

### Factors determining provider choice

#### Financial situation

“If you go down in the water, you will find the crocodile, if you go on land, you will find the tiger” one respondent mentioned during an interview. He meant that as a patient you always have to pay, regardless of the type of health provider you choose to visit. The financial situation of the patient is an important factor with regards to treatment choice, as families in Chhaeb and Chey Saen districts often severely indebt themselves when confronted with severe illnesses. The perceived severity of the illness and the perceived vulnerability of the patient will have a strong influence on their decision: the more severe the illness or the younger the patient, the more money families tend to invest in finding effective treatment.

*I have spent millions each year to get injections. For several years I had no energy and was [often] sick, so I went to the private practitioner. [I was said to have] ‘roh*-*leak tom rong nom’ (i.e. kidney inflammation) and ‘roh*-*leak dai sbon’ (i.e. uterus inflammation). I bought a lot of medicine to take, injections, and ‘serum’ (IV fluid therapy) too, but [I] could not see any improvement. My family never experienced nice food and a happy family life [during these years], because [I] was always in need of money to pay [the treatment costs]. [This went on] for three years, until [I] went to see an indigenous healer in Stung Treng. It was there I could recover, until today.* (Respondent in a focus group discussion with female farmers)

Although the malaria treatment itself is subsidized and therefore free of charge in the public health sector, consultation fees range from 1000 riel (0.25 USD) to 40,000 riel (10 USD) and are required to be paid up front, which can constitute a major financial challenge. Although multiple informants confirmed to have an ID Poor Card (related to the Health Equity Fund which exempts those who are registered as ‘poor’ from service fees), many informants complained that the criteria for selecting ‘poor’ families were not transparent and seemed unfair.

### Accessibility

The financial constraints families face in case of illness are not only linked to the cost of the provided treatment, but also to the transport costs to cover the distance to the health provider. While treatment at a nearby private ‘village doctor’ may be more expensive than at a distant public health centre, the total cost of going to the health centre is still higher if you take into account the transport costs. If it is not possible to go by foot or bicycle—because of the illness or the large distance—people tend to go by motorbike, two-wheel tractors, mini-van or boat, for which they usually need to pay the driver or the gasoline costs. These obstacles to access appropriate health care occur particularly in villages that do not have a public health facility nearby.

*R1: [When we are] sick, it is not always the case that we go to the health centre right away. Mostly [we] try first to find ‘tnam’ (i.e. biomedicines) from here and there, to swallow. [We] are unwilling to go [to the health centre], as it is far away and [to get there] it costs money too.*

*R2: [It’s] not a matter of unwillingness, but [we just] don’t have money.* (Two respondents in a focus group discussion with female farmers)

### Trust

Trust is often linked to the reputation of the health provider or past experiences people had with the perceived quality of the services, as well as the nature of the relationship between patient and provider. As many informants complained about the public health facilities due to bad reception, long waiting times, low quality of the services low, lacking equipment, and perceived ineffective treatments, qualitative data indicate little trust in public health facilities. The reportedly unfriendly and indifferent attitudes of health centre staff proves a barrier to attend public health centres, as exemplified by the following quotes.

*When there is no NGO (‘angkar’) nearby, the health centre staff ignores us, they pay no attention to us. […] It’s like a father hitting his child; the child does not have any right. They are the father; whatever they say, [people] have to believe it.* (Interview with a middle aged male farmer).

*I didn’t trust [them]… I was really unwell, but [they] didn’t do anything. […] I was waiting a half morning, until almost noon, and still [they] didn’t receive me. [At the end, they] just gave me one pill of medicine. [They] didn’t do anything.* (Interview with a middle aged female farmer)

Moreover, due to the frequent absence of health centre staff, waiting times are considered too long, making people fear their illness will deteriorate to a point where medical care is too late. In contrast, families will seldom lose trust in local indigenous healers in case of treatment failure, as they form an integral part of the Khmer health cosmology. Biomedical practitioners, however, continually have to earn people’s trust by giving good service and producing immediately observable improvements in the patient’s condition.

### Congruence between aetiology and treatment

Sorcery can only be treated by counter-sorcery measures. Biomedical medicines are considered powerless and ineffective as long as the spiritual affliction has not been neutralized. “It is like pouring water on the duck’s neck”, as one informant put it, by which he meant that it is simply pointless to receive biomedical treatment as long as the spiritual or magical affliction is not neutralized. People will usually consult a *kru khmer* to check if something ‘invisible’ (*vea mean ey na*) is causing the illness, after which the *kru khmer* restores the disrupted social and spiritual relations. If the neutralization of the spiritual or magical affliction is successful, biomedical treatment will be considered effective again. If the sick person does not fully recover after his visit to the *kru khmer*, a return to the medical practitioner (*kru pet*) to continue the biomedical treatment is in order. Many respondents do not recur to only one specific health provider, but often combine different options of treatment during the same illness episode through a serial process of trial and error that is guided by the observable improvements in the patient’s condition.

## Discussion

In 2014, the WHO recommended the upgrading of Preah Vihear province to tier 1 due to the emerging resistance to artemisinin. This study shows that the containment of parasite resistance in this area is challenged by limited access to the first line treatment due to poverty, structural barriers and varying illness perceptions.

Illness behaviours are acquired by socialization and shared collective experience, and as such are culturally shaped [25]. The delay of malaria treatment seeking in Preah Vihear province is therefore partly linked to Cambodian perceptions of illness which focus on single symptoms or specific body parts [22, 26]. Congruent with the perceived symptoms, the sick person and his or her social network will self-diagnose, identifying one or multiple illnesses at the same time. Each of these symptom-based illnesses may then imply different patterns of health-seeking behaviour and treatment choice, explaining why families will rarely suspect *krun chan* (malaria) from the start and fail to seek prompt and appropriate care in case of malaria. ‘Fever’ can be subdivided in a variety of bodily experiences for which different Khmer terms exist, suggesting different diagnoses and patterns of health-seeking behaviour. Finally, the different Khmer words that may refer to malaria—*krun chan, krun che*, *krun vivak, krun andeuk*—demonstrate that there is no uniform pattern of self-diagnosis and associated health-seeking behaviour, nor a single malaria concept that health education messages could identify with.

However, rather than correlating the factors ‘cultural beliefs’ or ‘illness perceptions’ directly to the delay in seeking appropriate care, this study stresses the importance of understanding these findings within a local context where poverty dictates pragmatic health-seeking decisions. Rural Cambodians often lack immediately available financial resources to meet the costs that come with ‘appropriate’ medical care, either in the public or the private sector. Self-diagnosis and home-treatment are therefore not just ‘Cambodian customs’, they are often the only choice for families, as they cannot initially differentiate between the similar signs of the many infectious diseases endemic in the area and do not have ready cash to deal with the costs of potentially minor ailments, including the cost of travelling the distance to the health provider. Moreover, the lack of trust in public health providers may further influence the treatment choice of families. Many informants in Preah Vihear complained about the low quality and ‘unofficial’ high cost of the public services that are meant to provide the ‘appropriate’ treatment. Ovesen and Trankel mention in this sense also the “mental distance” to the public health facility [22]. These structural factors should therefore be considered the genuine obstacles in accessing appropriate malaria treatment [27].

Frequent reliance on treatments bought from untrained drug vendors or village doctors in Cambodia, instead of the appropriate treatments available in the public sector, have been reported in other studies as well [17, 19, 20, 22]. A wide range of potentially substandard biomedical medication is available at local village shops, drugstores or at the nearby private ‘village doctor’ (*pet phum*), where prior consultation or prescriptions are not required. Private cabinets are often serviced by the same people that work as health staff in the public health centres, insinuating a more systemic problem in the Cambodian health care system [17, 22]. Such private practitioners will listen to specific requests, giving some level of control over the choice of medicine to the patients and their families. However, reflecting findings from previous studies in Cambodia, these health-seeking patterns are not fixed [19, 20]. Often different treatment modalities are combined at the same time: the indigenous healer may be visited while the patient is still taking well-known medicines such as paracetamol (*para*), amoxicillin (*amok*) and ampicillin (*ampi*), or even malaria combination therapies and monotherapies such as quinine tablets, injectable quinine, chloroquine and tetracycline, which can all be found at local level. These are then often again combined with intravenous fluids and injections, which are popular options in the private sector that are perceived to be more powerful than pills and speed up recovery [19, 28]. However, the quality of drugs in the informal private sector cannot be assured [17, 29, 30], making these practices potentially dangerous in light of the mounting resistance to single and combination drug treatments for malaria.

## Conclusions

In the context of current malaria elimination goals, the structural barriers (financial situation, lack of trust, problems of accessibility) and methods of self-diagnosis (symptom-based, divination, intra- and extra familial discussions) and self-treatment (herbal treatments, manual therapies and private sector medicines) may cause delay in the appropriate malaria treatment-seeking required for creating the conditions necessary to contain the drug resistant parasite strains in Preah Vihear province. Each untreated or mistreated case could potentially contribute to maintaining a parasite reservoir and further spread resistant strains.
